# A robot-based behavioural task to quantify impairments in rapid motor decisions and actions after stroke

**DOI:** 10.1186/s12984-016-0201-2

**Published:** 2016-10-10

**Authors:** Teige C. Bourke, Catherine R. Lowrey, Sean P. Dukelow, Stephen D. Bagg, Kathleen E. Norman, Stephen H. Scott

**Affiliations:** 1 Centre for Neuroscience Studies, Queen’s University, Kingston, ON Canada; 2 Department of Physical Medicine and Rehabilitation, Queen’s University, Kingston, ON Canada; 3 School of Rehabilitation Therapy, Queen’s University, Kingston, ON Canada; 4 Department of Biomedical and Molecular Sciences, Queen’s University, Kingston, ON Canada; 5 Department of Clinical Neurosciences, Hotchkiss Brain Institute, University of Calgary, Calgary, AB Canada

**Keywords:** Stroke, Assessment, Cognitive impairments, Attention, Inhibition, Neglect

## Abstract

**Background:**

Stroke can affect our ability to perform daily activities, although it can be difficult to identify the underlying functional impairment(s). Recent theories highlight the importance of sensory feedback in selecting future motor actions. This selection process can involve multiple processes to achieve a behavioural goal, including selective attention, feature/object recognition, and movement inhibition. These functions are often impaired after stroke, but existing clinical measures tend to explore these processes in isolation and without time constraints. We sought to characterize patterns of post-stroke impairments in a dynamic situation where individuals must identify and select spatial targets rapidly in a motor task engaging both arms. Impairments in generating rapid motor decisions and actions could guide functional rehabilitation targets, and identify potential of individuals to perform daily activities such as driving.

**Methods:**

Subjects were assessed in a robotic exoskeleton. Subjects used virtual paddles attached to their hands to hit away 200 virtual target objects falling towards them while avoiding 100 virtual distractors. The inclusion of distractor objects required subjects to rapidly assess objects located across the workspace and make motor decisions about which objects to hit.

**Results:**

As many as 78 % of the 157 subjects with subacute stroke had impairments in individual global, spatial, temporal, or hand-specific task parameters relative to the 95 % performance bounds for 309 non-disabled control subjects. Subjects with stroke and neglect (Behavioural Inattention Test score <130; n = 28) were more often impaired in task parameters than other subjects with stroke. Approximately half of subjects with stroke hit proportionally more distractor objects than 95 % of controls, suggesting they had difficulty in attending to and selecting appropriate objects. This impairment was observed for affected and unaffected limbs including some whose motor performance was comparable to controls. The proportion of distractors hit also significantly correlated with the Montreal Cognitive Assessment scores for subjects with stroke (*r*_*s*_ < = − 0.48, *P* < 10^−9^).

**Conclusions:**

A simple robot-based task identified that many subjects with stroke have impairments in the rapid selection and generation of motor responses to task specific spatial goals in the workspace.

## Background

Moving and interacting in the world requires rapid processing of the visual environment to identify potential motor goals, select a movement and finally move in a timely manner. For example, when packing groceries, we must decide where to put items based on their shape, size, fragility and other features. The selection, planning and execution of motor actions must be done rapidly to keep pace with the flow of groceries from the cashier.

There is growing evidence that sensory feedback is rapidly integrated into motor decisions [1–3]. Sensory feedback is integrated with higher-level behavioural goals to make rapid decisions on how to move and interact in the environment. Selective attention refines spatial representations of the environment into potential movement targets [1, 4]. The choice between these internal representations is then based on ‘decisional factors’ [1]. One such factor is the recognition of combinations of visual features and their behavioural relevance [1, 5, 6]. Thus, the sensorimotor system rapidly integrates information on the environment to guide motor decisions.

Another important aspect of voluntary motor control is the ability to inhibit a motor action [7]. When instructed, it is very automatic to simply reach towards spatial targets as they appear in the workspace [8]. In contrast, it can be hard to avoid reaching towards a target when instructed to move in the opposite direction. In this anti-reach condition, subjects can make erroneous initial motor responses to the spatial goal and are delayed in moving in the opposite direction. This task requires the voluntary override of an automatic response to reach towards the target and involves many brain areas including frontal and parietal cortex [7, 9–12]. This ability can be impaired in persons with stroke [12], mild cognitive impairment [13], Alzheimer’s Disease [14], and a history of concussion [15], Thus, successful voluntary motor control involves processing sensory feedback not only to select motor actions but also to avoid making others.

Post-stroke disability stems from a variety of motor, sensory, and/or cognitive deficits [16]. The ability to pack groceries described above highlights that impairments in these functional tasks may reflect not only motor impairments but also cognitive impairments. When driving, one must quickly decide on actions to apply pressure to the brake or accelerator pedals, or turn the wheel based on information from street signs, traffic signals, other traffic, and pedestrians. However, neuropsychological tests or cognitive screening tools generally separate motor and cognitive assessments – the latter often requiring verbal or written responses – and typically do not impose time limits to perform the tasks [17, 18]. Few neuropsychological assessments focus on rapid motor decisions beyond simple reaction time tests [18, 19], or timed cognitive tasks such as trail making [20], even though complex and time sensitive demands are often required for everyday activities.

Furthermore, many standard assessments of post-stroke functioning have problems of subjectivity, coarse ordinal scales, criteria-based scoring, and lack of responsiveness (including floor and ceiling effects) [21]. Thus, we developed a novel approach of using a robotic assessment to provide objective, continuous measures of performance that are compared to a normative model of healthy control performance.

We recently used an object hit task to quantify simultaneous upper limb bimanual sensorimotor performance [22]. Although this task quantified rapid motor skills, decisional processes required to perform the task were limited to identifying the trajectory of an object and selecting a limb to hit the object. As all objects were targets in this task, it did not require cognitive processes related to attending to object qualities (rather than just spatial location) to select a motor action nor require inhibiting inappropriate motor responses. These processes can be impaired following stroke [9, 23, 24].

The goal of the present study was to develop a task that examined rapid motor skills with both arms that also required greater cognitive processing. We developed a variant of the object hit task [22] by requiring subjects to hit 2 possible targets while avoiding all other objects in the workspace. We hypothesize that individual subjects with stroke will be impaired in enacting or inhibiting a motor response to a potential target based on sensory feedback of the object’s features and their relevance to the ongoing task. The performance of subjects with stroke was compared to a large cohort of non-disabled control subjects.

Clinically, knowledge of impairments in these more complex visuomotor skills can guide novel rehabilitation strategies to regain the ability to rapidly process sensory information for motor actions. As well, it may help to identify if individuals should return to more complex daily activities such as driving.

## Methods

### Subject information

Participants included patients recruited from Providence Care (St. Mary’s of the Lake Hospital, Kingston, ON), the Dr. Vernon Fanning Centre and Foothills Hospital (Calgary, AB). Prospective subjects were excluded if they had other significant neurologic diagnoses (e.g., Parkinson’s disease), acute medical illness, and/or ongoing upper extremity musculoskeletal injuries. Subjects were also excluded if they appeared fatigued, reported pain associated with attempting robotic assessments or reported pain during clinical testing on strength or range of motion that would be relevant to the robotic task. Non-disabled control subjects were recruited from the Kingston, ON and Calgary, AB communities. This study was approved by the Queen’s University Health Sciences and Affiliated Teaching Hospitals Research Ethics Board (#ANAT-024-05) and the University of Calgary’s Conjoint Health Research Ethics Board (#22123) and subjects provided informed consent.

### Experimental setup

Details of the robotic set-up have been reported previously [25, 26]. Briefly, the behavioural task was performed using a bimanual exoskeleton robot which measures limb motion (KINARM, BKIN Technologies Ltd, Kingston, ON, Canada). Participants sat in a modified wheelchair base, and their arms were fitted in supports permitting movement in the horizontal plane. Arm supports were adjusted such that the robot’s linkages aligned with the subject’s elbows and shoulders. Subjects received visual feedback from a virtual reality system which displayed fingertip position and virtual objects in the same plane as arm motion via a two-way mirror. Direct vision of the hands and arms was occluded.

### Behavioural task

**Fig. 1:**
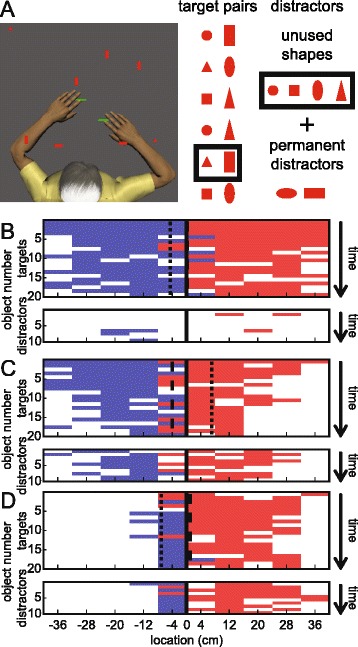
Task details and exemplar subjects. **a** Screenshot of a subject performing the task. Objects included 2 target shapes (chosen from 6 pair variants) and 6 distractor shapes (4 are shapes used as targets in other task variants and 2 were always distractors). **b** Task performance summary of a 62 year old right-handed male control subject. Y axes are number of targets (*top*) or distractors (*bottom*) dropped from each bin (*X axis*). Hits with the left hand are blue areas and hits with the right hand are red areas. Missed objects are the white areas. The top of each plot represents the beginning of the task, and the bottom represents the end. Hand transition and miss bias are indicated with dashed and dotted lines (respectively). **c** Performance of a 65 year old right-handed, right-affected male subject 5 days post-stroke. **d** Performance of a 63 year old right-handed male subject 8 days post-stroke. Subject was left-affected and had a BIT score of 67 (indicative of visual neglect)

Subjects were assessed in an object hit and avoid task (Fig. 1a), which is based on a previous object hit task [22]. At the beginning of the task, subjects were presented two shapes on the screen. Subjects were instructed to hit these two shapes (‘targets’) away from them and avoid all other shapes (‘distractors’). Subjects could use both hands which were represented by horizontal paddles. Both target objects and distractor objects dropped from one of 10 locations along the top of the screen 8 cm apart (virtual bins). A total of 30 objects (20 targets and 10 distractors) were released at each bin (200 targets and 100 distractors total). Objects were released from all 10 bins before a bin was reused. Objects dropped at an increasing rate following the equation:

$$ \mathrm{Drop}\ \mathrm{Rate} = 0.5\ \mathrm{objects}/\mathrm{second} + \left[0.025\ \mathrm{objects}/{\mathrm{second}}^2 \times \left(\mathrm{time}\left(\mathrm{s}\right)\right)\right] $$

The maximum number of objects possible to appear on the screen simultaneously increased from 1 to 16 over the course of the task. The speed of the objects moving towards the subject was 50 to 100 % of maximum drop speed, which increased following the equation:

$$ \max\ \mathrm{drop}\ \mathrm{speed} = 15\ \mathrm{cm}/\mathrm{s} + \left[0.3\ \mathrm{cm}/{\mathrm{s}}^2 \times \left(\mathrm{time}\left(\mathrm{s}\right)\right)\right] $$

Thus targets moved at ~10 cm/s in the beginning of the task and increased to ~50 cm/s by the end of the task. Position of the objects and hand position were recorded at 200 Hz. The task took just over 2 min to complete.

One of 6 task variants were used with varying shapes designated as targets and distractors. Target pairs had similar width but were always different heights and different classes of shapes (Fig. 1a). Distractors consisted of the remaining unused shapes (shapes used as targets in other task variants), as well as two wider shapes.

Every effort was made to ensure subjects understood the task instructions. Operators usually obtained verbal confirmation that they understood which targets to hit when showing the target objects before starting the task. Reminders to hit the specific target shapes and avoid all others would be given early in the task, especially if there seemed to be confusion with similar distractor shapes (for example tall target rectangle vs. wide rectangle distractor). As well, targets hit by a paddle were knocked away and haptic feedback of the contact was provided by the robot [22], whereas distractors simply passed through the paddle to provide immediate feedback that it was a distractor.

### Data processing

Data were analyzed using MATLAB (Mathworks Inc., Massachusetts). Hand speed was filtered using a sixth-order double-pass Butterworth filter (cutoff frequency 10 Hz).

### Tasks parameters

We used 14 metrics to quantify task performance in order to characterize a diverse range of sensory, motor and cognitive functions examined in this task. Most of the parameters paralleled the metrics in our previously published object hit task [22], a simpler version of this task in which there were no distractors. As well, a few parameters were added or modified to capture the addition of distractors in the present task.

Global Performance was evaluated using five parameters:

1. Targets hit: The number of target objects hit away from the body.
2. Distractors hit: The number of distractor objects hit.
3. Objects hit: The number of objects hit (target hits + distractor hits).
4. Distractor proportion: Distractors hit divided by objects hit.

In the case of multiple hits for the same object, the first hit is used to determine which hand/paddle hit the ball.

5. Object processing rate (objects/second): The rate of correctly processed objects (number of targets hit + distractors missed per second) at 80 % of task completion. The rate of correctly processed objects was determined at every time step (every 0.005 s) from the time the first object was hit or left the screen to the time the 240^th^ object (80 % of task complete) was hit or left the screen. To filter this signal, we convolved the rate with a Gaussian window (MATLAB function normpdf). From this rate signal, the optimal growth curve (y = <max height > *(1-exp(−(<curvature>)* < data>))) [27, 28] for the data was calculated and the value of this curve when 80 % of the task was completed was used to approximate the maximal object processing rate for each individual subject. The rate was taken at 80 % of task complete so that performance was at or near maximum, but not at 100 % as the ratio of distractor object dropping statistically increased at the end of task. This is because there is always a 66 % chance of dropping a target object, but objects are sampled without replacement, leading to the statistical scenario of running out of target objects and only being able to drop distractor objects from a given bin at the end of the task.

All other parameters were defined in the same way as the object hit task [22].

Spatial and Temporal Performance

6. Miss bias: Spatial position quantifying the extent to which the number of target misses deviates from being equally distributed on either side. Computed as sum of target misses in each bin (m), multiplied by the bin position (x), and then divided by the total number of target misses (sum(mx)/sum(m)). Given that the centre of the bins is x = 0, the greater the mean location of misses deviates from 0, the more misses were on the left or right side of the workspace (dependent on whether the miss bias is negative or positive, respectively).
7. Hand transition: Spatial transition point in subject’s hand preference for hitting targets. This is the mean of the right hand’s and the left hand’s weighted means of their respective target hit distributions. The weighted mean of each hand only includes target hits by that hand in bins where both hands have been used to hit targets (overlapping bins) and one additional bin beside the overlapping bins (where that hand has been used to hit targets). If bins do not overlap, only the leftmost bin with target hits from the right hand and the rightmost bin with target hits from the left hand are used in the weighted means.
8. Median error (% of targets): Point in time when subjects missed half of the target objects that they missed over the entire task.

Hand Specific Performance

9. Movement Area: The areas of space used by each hand during the task. Computed as the area of the convex hull- a complex polygon which captures the boundaries of the movement trajectories of each hand [22, 29]. Calculated for each hand separately.
10. Hand speed: The average hand speed calculated from each time step (5 ms) over the course of the task. Calculated for each hand separately.
11. Hand bias hits: The difference between the number of target hits with the right hand and the number of target hits with the left hand divided by the total number of target hits.
12. Hand selection overlap: The number of times successive target hits from a given bin were with different hands divided by the total number of target hits.
13. Hand movement bias area: Difference in movement area of the right and left hands divided by the sum of the movement area of the right and left hands.
14. Hand bias speed: The difference in mean hand speed of the right and left hands divided by the sum of the mean hand speed of the right and left hands.

### Statistical analysis

Performance of control subjects was analyzed for any effects of age, sex, or handedness. Control values were age-regressed and Box Cox transforms were used to normalize control distributions when necessary [30, 31]. Control parameter values were then assessed for any effect of sex or handedness, and values subdivided into respective categories if effects were significant, and age regressed and Box Cox transformed again if necessary. All parameter values were converted to z-scores of the model to allow for comparison across all subjects (because age, sex, and handedness are now accounted for in the model). Individual subjects with stroke were defined as having impaired performance on a task parameter, when their z-score was >1.65 or < −1.65 for one tailed tests, or > |1.96| for two tailed tests.

A subset of subjects was assessed a second time by a different operator within 7 days of their initial assessment. An intraclass correlation was used to determine interrater reliability (significant if *P* < 0.05, acceptable if ICC > 0.8).

### Clinical assessments

Subjects with stroke were evaluated by a trained physician, physiotherapist, or occupational therapist using a number of standardized clinical assessments. Both arms were assessed using the Chedoke-McMaster Stroke Assessment (CMSA) [32] to determine arm function. The CMSA is based on Brunnstrom’s stages of motor recovery post-stroke [33]. Subjects were broadly categorized as “Left-Affected” (LA) or “Right-Affected” (RA) depending on the clinically most affected side of the body. Elbow flexor spasticity was measured by the Modified Ashworth Scale, which categorizes the amount of resistance produced by the arm in response to passively moving it through its range of motion [34]. Functional abilities were measured with the Functional Independence Measure (FIM), which has both a motor and cognitive component [35]. The conventional subtests of the Behavioural Inattention Test (BIT) were used to screen for deficits in spatial attention [36]. Subjects with stroke who scored <130 on the BIT were defined as having visuospatial neglect and were analyzed separately (neglect subjects). Subjects were also screened for cognitive deficits using the Montreal Cognitive Assessment (MoCA) [37]. The handedness of controls and subjects with stroke was determined by the Modified Edinburgh Handedness test [38].

## Results

### Subject demographics and clinical information

Table 1 shows the demographic information and clinical scores for the 157 subjects with stroke and 309 control subjects. The majority of subjects with stroke were assessed early, with only 17/157 subjects being assessed >28 days of their stroke. Exclusion of these 17 subjects did not substantively alter the present results. Subjects with stroke were usually assessed either on the same day (n = 90) or within 1 day (n = 40) of the robotic assessment. Some subjects with stroke were assessed within 2–4 days (n = 18) and a few within 5–10 days (n = 9). Twenty eight subjects with stroke displayed visual neglect as indicated by scores of <130 on the BIT. These subjects were analyzed separately to assess differences in the patterns of task performance with stroke and visual neglect.

**Table 1:** Demographic information of subjects included in the experiment

Measure	Stroke (n = 157)								Controls (n = 309)
Age in years^a^	64 (25–90; 13.8)								53 (18–93; 19.4)
sex (male/female)	102/55 subjects								138/171 subjects
handedness (L/R/M)^b^	10/147/0 subjects								30/279/0 subjects
time since stroke^a^	11 (1–49; 11.2) days								-
affected arm (L/R)	94/63 subjects								-
FIM- motor subscore^cd^	71 (22–91)								-
FIM- total score^cd^	102.5 (46–126)								-
MoCA^e^	24 (10–30)								-
BIT^c^	141 (64–146)								-
lesion location (number of subjects)	C	SC	C + SC	Cb	Br	Cb + Br	Mx	Uk	-
	44	48	44	3	8	1	3	4	
	LA (n = 94)				RA (n = 63)				
ischemic/hemorrhagic/both	85/9/0 subjects				55/8/0 subjects				-
BIT < 130	22 subjects				6 subjects				-
visual field deficit	16 subjects				7 subjects				-
CMSA- arm subscore^f^									
Affected arm	[5 13 14 4 19 14 24] ^g^				[5 8 9 5 13 7 15] ^h^				-
Unaffected arm	[0 0 0 0 8 22 63] ^g^				[0 0 0 0 1 14 47] ^h^				-
Affected hand	[9 3 10 8 30 16 16] ^i^				[4 7 6 7 12 11 15] ^h^				-
Unaffected hand	[0 0 0 0 1 32 59] ^i^				[0 0 0 0 2 18 42] ^h^				-
	left		right		left			right	
Modified Ashworth^j^	[76 10 1 6 0 0]^g^		[61 1 0 0 0 0]^g^		[91 2 0 0 0 0]^h^			[48 8 4 0 2 0]^h^	

*Abbreviations: L/R/M* (left/right/mixed)*, FIM* (functional independence measure)*, MoCA* (Montreal cognitive assessment), *BIT* (behavioural inattention test), *LA* (left affected), *RA* (right affected), *C* (cortical), *SC* (subcortical), *C + SC* (cortical + subcortical), *Cb* (cerebellar), *Br* (brainstem), *Cb + Br* (cerebellar + brainstem), *Mx* (mixed), *Uk* (unknown), *CMSA* (Chedoke-McMaster Stroke Assessment)

Legend:^a^ median (min-max; standard deviation).^b^ Handedness as determined by the Edinburgh Handedness Test.^c^ median (min-max).^d^ n = 156.^e^ n = 152.^f^[n1 n2 n3 n4 n5 n6 n7] corresponds to the number of subjects with CMSA subscores of [1 2 3 4 5 6 7].^g^ n = 93.^h^ n = 62.^i^ n = 92.^j^ [n1 n2 n3 n4 n5 n6] corresponds to the number of subjects with Modified Ashworth scores of [0 1 1+ 2 3 4] for elbow flexion

### Exemplar subjects

Figure 1b-d displays the distribution of target and distractor hits and misses for a control subject and two subjects with stroke. The control subject (Fig. 1b) was very effective in hitting targets (136/200) and avoiding distractors (94/100). Control subjects gradually missed more targets, especially lateral ones, as task difficulty increased. The RA subject with stroke (Fig. 1c) hit fewer targets (117/200) and more distractors (47/100) than the control. The LA neglect subject (Fig. 1d) hit even fewer targets (83/200) and a similar amount of distractors (40/100). This subject also hit very few objects with their left hand and very few on the left side of the workspace.

### Impairments identified using the robot-based task

**Fig. 2:**
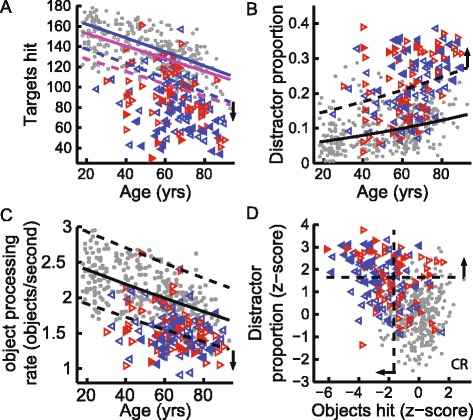
Global Performance in Task Parameters. **a** Scatter plot of age versus target hits. Performance of male and female controls is shown by filled and empty grey markers, respectively. Performance of subjects with stroke is shown by the leftward and rightward pointing triangles representing left-affected and right-affected subjects, respectively. Triangle markers are filled if subject also had a BIT score <130 indicative of visual neglect. Age normative model is shown by the blue and magenta lines representing the median (*solid lines*) and cutoff (*dashed lines*) z-score for male and female control subject performance distribution (respectively) according to the model. The black arrow indicates which side of the cutoff score corresponds with subjects being impaired on the particular parameter. **b** Scatter plot of age versus distractor proportion. Performance of control subjects is shown by the filled grey markers. Age normative model is shown by the median and cutoff z-score of control subject performance distribution according to the model. **c** Scatter plot of age versus estimated maximum object processing rate. **d** Scatter plot of object hits versus distractor proportion. Values have been converted to z-scores based on the normative models. Dashed lines represent the cutoff used to indicate impairment in each parameter. The control performance range is the quadrant indicated by the ‘CR’

Each parameter classified a varying number of subjects with stroke as impaired (Table 2). Target hits identified the largest number of subjects as impaired. The number of targets hit by controls depended on age and sex (Fig. 2a). Subjects with stroke were considered to be impaired in targets hit if their performance fell below the 5 % level performance of controls after correcting for age and sex. Overall, 78 % of LA subjects (left-affected subjects with stroke), 68 % of RA subjects (right affected subjects with stroke), and 96 % of neglect subjects (subjects with stroke and visual neglect) were impaired in targets hit. Similarly, age effects were found for objects hit. In total, 64 % LA subjects, 51 % RA subjects, and 86 % of neglect subjects hit less objects than the lower cutoff of the age normative model.

**Table 2:** Task performance, interrater reliability, and clinical correlations. Task parameter sensitivity is defined by the corresponding z-score cutoff range

			Subjects with stroke (% impaired)			Interrater reliability
Parameters (normative models)		z-score cutoff	BIT > =130		BIT < 130	Intraclass correlation (*P*)
			LA	RA		
		Global performance				
Target hits		<−1.645	78	68	96	0.93 (2×10^−11^)
Distractor hits		>1.645	6	23	21	0.80 (2×10^−6^)
Object hits		<−1.645	64	51	86	0.89 (2×10^−9^)
Distractor proportion		>1.645	39	51	79	0.90 (2×10^−9^)
Object processing rate		<−1.645	42	51	69	0.83 (2×10^−7^)
		Spatial and temporal performance				
Miss bias		>|1.96|	25	0	21	0.54 (0.004)
Hand transition		>|1.96|	32	33	57	0.24 (0.11)
Median error		<−1.645	51	53	82	0.55 (0.002)
		Hand specific performance				
Hand bias (hits)		>|1.96|	67	69	75	0.89 (5×10^−9^)
Hand selection overlap		<−1.645	21	23	32	0.61 (8×10^−4^)
Total hand bias area		>|1.96|	50	53	71	0.80 (5×10^−7^)
Hand bias speed		>|1.96|	63	64	86	0.93 (5×10^−11^)
object hits	left	<−1.645	85	14	89	0.90 (2×10^−10^)
	right	<−1.645	29	74	54	0.78 (4×10^−6^)
Distractor proportion	left	>1.645	59	39	96	0.93 (7×10^−11^)
	right	>1.645	26	67	61	0.84 (1×10^−7^)
Total hand area	left	<−1.645	60	14	68	0.91 (3×10^−10^)
	right	<−1.645	25	40	32	0.81 (3×10^−7^)
hand speed	left	<−1.645	68	14	86	0.89 (4×10^−9^)
	right	<−1.645	21	54	50	0.84 (2×10^−7^)
Parameters impaired		95 % (>5)	79	79	96	0.96 (1×10^−14^)

*Abbreviations: BIT* (behavioural inattention test), *LA* (left-affected subject), *RA* (right-affected subject)

Interrater reliability is shown by the intraclass correlation and corresponding *p*-values

Distractor proportion identified more individual subjects with stroke as impaired (39 % LA, 51 % RA, 79 % neglect; see Fig. 2b and Table 2) compared to distractors hit (15 % of subjects with stroke impaired). Object processing rate was also a parameter that identified a large proportion of subjects with stroke (Fig. 2c): controls mostly had a processing rate between 1.5 to 3 objects per second, whereas the object processing rate of most subjects with stroke was below 2.

Subjects with stroke who hit fewer objects also tended to hit a higher proportion of distractors (Spearman correlation; controls: *r*_*s*_ = 0.16, *P* = 0.006; subjects with stroke: *r*_*s*_ = −.33, *P* = 3×10^−5^). Twenty nine percent of subjects with stroke displayed impairments in both object hits and distractor proportion (Fig. 2d, upper left quadrant). In contrast, 29 % of subjects with stroke had impairments in only object hits (Fig. 2d, lower left quadrant) and 16 % had impairments in only distractor proportion (Fig. 2d, upper right quadrant). All neglect subjects were impaired in at least one of these two parameters, and 64 % were impaired in both parameters.

**Fig. 3:**
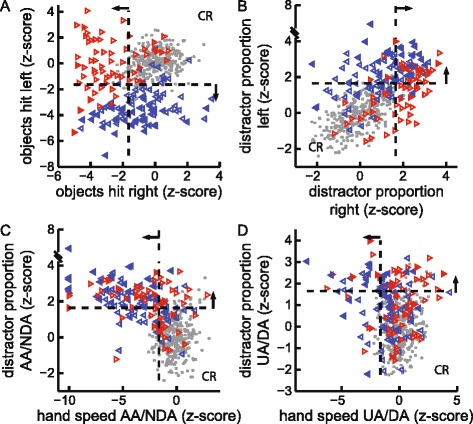
Hand Specific Performance in Task Parameters. **a** Scatter plot of object hits (z-score) with the right versus the left hand. Symbols same as Fig. 2. **b** Scatter plot of distractor proportion with the right versus the left hand. **c** Scatter plot of hand speed versus distractor proportion with the affected arm (AA) of subjects with stroke and non-dominant arm (NDA) of control subjects. **d** Scatter plot of hand speed versus distractor proportion with the unaffected arm (UA) of subjects with stroke and dominant arm (DA) of control subjects

Almost all subjects with stroke (92 %) hit fewer objects with their affected arm than with their unaffected arm (Fig. 3a). Similarly, 77 % of subjects with stroke showed a greater distractor proportion with their affected arm than with their unaffected arm (Fig. 3b). Overall, 57 % of LA subjects, 60 % of RA subjects, and 50 % of neglect subjects had impaired object hits with their affected arm only.

Motor and distractor-related impairments in performance of the affected arm commonly co-occurred. Of the subjects whose affected arm was impaired in distractor proportion, the same arm was usually also impaired in objects hit (97 % LA, 81 % RA) and/or hand speed (85 % LA, 63 % RA) (Fig. 3c). Distractor proportion was also negatively correlated with the number of object hits by the affected arm of subjects with stroke (*r*_*s*_ = −0.58, *P* < 10^−10^). Although this would be expected, a negative correlation was not observed in the non-dominant arm of controls (*r*_*s*_ = 0.15, *P* = 0.008).

In contrast, this coupling of motor and distractor-related impairments was less common for the unaffected arm. For the subjects with stroke who had impaired distractor proportion with their unaffected arm, the majority did not have impaired object hits (47 % LA, 82 % RA) or hand speed (58 % LA, 75 % RA) with the same arm (Fig. 3d). The correlation between distractor proportion and objects hit was weaker for unaffected arm performance of subjects with stroke (*r*_*s*_ = −0.15, *P* = 0.07). A negative correlation was not observed for the dominant arm of controls (*r*_*s*_ = 0.18, *P* = 0.001).

Neglect subjects who had impaired distractor proportion with their affected arm were usually impaired in hitting objects and/or hand speed with the same arm (100 and 92 % impaired in both parameters, respectively). Impairments in distractor proportion for their unaffected arm were less likely to co-occur with impairments in objects hit and/or hand speed with that arm (36 and 36 % impaired in both parameters, respectively).

We aggregated the number of task parameters that each subject was impaired in (Table 2). Most subjects with stroke (82 %) were impaired in more task parameters than 95 % of controls (>5 parameters). This included all but one subject with neglect.

### Correlations with standard clinical assessments

**Fig. 4:**
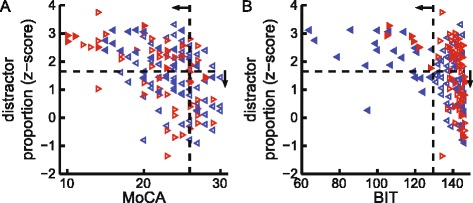
Clinical correlations with task performance. **a** Scatter plot of Montreal Cognitive Assessment (MoCA) scores versus overall distractor proportion. Symbols same as Fig. 2. **b** Scatter plot of Behavioural Inattention Test (BIT) scores versus overall distractor proportion

Task parameter measures were compared to scores on the FIM, MoCA, and BIT (Table 3). Object hits showed moderate correlations with BIT (*r*_*s*_ = 0.40, *P* = 2×10^−7^) and FIM scores (*r*_*s*_ = 0.45, *P* = 3×10^−9^) and weak correlations with MoCA scores (*r*_*s*_ = 0.23, *P* = 0.004). Distractor hits displayed modest correlations with MoCA (*r*_*s*_ = −0.31, *P* = 1×10^−4^), but distractor proportion displayed moderate correlations with BIT (*r*_*s*_ = −0.43, *P* = 2×10^−8^), FIM (*r*_*s*_ = −0.45, *P* = 4×10^−9^), and MoCA (*r*_*s*_ = −0.49, *P* = 2×10^−10^) (Fig. 4a,b). Out of the 36 % of subjects with stroke who passed the MoCA (scored > =26), 27 % of these subjects with stroke were impaired in distractor proportion. Object hits with the affected arm correlated better with the FIM (*r*_*s*_ = 0.51, *P* = 1×10^−11^) than the unaffected arm (*r*_*s*_ = 0.26, *P* = 8×10^−4^). Object hits with the unaffected arm showed a modest correlation with BIT (*r*_*s*_ = 0.30, *P* = 1×10^−4^) and a weak correlation with MoCA (*r*_*s*_ = 0.17, *P* = 0.04). Distractor proportion with the unaffected arm showed moderate correlations with BIT (*r*_*s*_ = −0.41, *P* = 9×10^−8^) and MoCA (*r*_*s*_ = −0.48, *P* = 4×10^−10^). MoCA scores correlated most strongly with overall and unaffected arm distractor proportion (*r*_*s*_ = <= − 0.48, *P* < 10^−9^). The number of parameters impaired was also moderately correlated with FIM (*r*_*s*_ = −0.61, *P* = 2×10^−17^) and BIT (*r*_*s*_ = −0.43, *P* = 3×10^−8^) scores.

**Table 3:** The relationship between task performance of subjects with stroke and Functional Independence Measure (FIM), Montreal Cognitive Assessment (MoCA), and Behavioural Inattention Test (BIT) scores is shown by the corresponding Spearman correlations

Parameters (normative models)		Spearman correlations *r*_*s*_ (*P*)		
		FIM total	MoCA	BIT
		Global performance		
Target hits		0.52 (3×10^−12^)	0.38 (1×10^−6^)	0.48 (2×10^−10^)
Distractor hits		−0.14 (0.09)	−0.31 (1×10^−4^)	−0.17 (0.03)
Object hits		0.45 (3×10^−9^)	0.23 (0.004)	0.40 (2×10^−7^)
Distractor proportion		−0.45 (4×10^−9^)	−0.49 (2×10^−10^)	−0.43 (2×10^−8^)
Object processing rate		0.39 (6×10^−7^)	0.27 (6×10^−4^)	0.33 (3×10^−5^)
		Spatial and temporal performance		
Miss bias		0.10 (0.22)	−0.08 (0.32)	0.20 (0.01)
Hand transition		−0.16 (0.05)	−0.11 (0.17)	−0.03 (0.72)
Median error		0.48 (3×10^−10^)	0.36 (6×10^−6^)	0.44 (8×10^−9^)
		Hand specific performance		
Hand bias (hits)		−0.01 (0.93)	0.12 (0.15)	−0.20 (0.01)
Hand selection overlap		0.30 (2×10^−4^)	0.11 (0.18)	0.10 (0.21)
Total hand bias area		0.02 (0.80)	0.15 (0.07)	−0.15 (0.06)
Hand bias speed		−0.01 (0.87)	0.13 (0.11)	−0.18 (0.02)
Object hits	Affected	0.51 (1×10^−11^)	0.24 (0.003)	0.38 (7×10^−7^)
	Unaffected	0.26 (8×10-4)	0.17 (0.04)	0.30 (1×10-4)
Distractor proportion	Affected	−0.45 (5×10^−9^)	−0.31 (8×10^−5^)	−0.36 (4×10^−6^)
	Unaffected	−0.37 (2×10^−6^)	−0.48 (4×10^−10^)	−0.41 (9×10^−8^)
Total hand area	affected	0.44 (6×10^−9^)	0.07 (0.43)	0.22 (0.005)
	Unaffected	0.14 (0.08)	0.17 (0.04)	0.29 (3×10^−4^)
hand speed	Affected	0.52 (3×10^−12^)	0.14 (0.09)	0.33 (2×10^−5^)
	Unaffected	0.24 (0.002)	0.16 (0.06)	0.35 (7×10^−6^)
Parameters impaired		−0.61 (2×10^−17^)	−0.31 (1×10^−4^)	−0.43 (3×10^−8^)

*Abbreviations: RH* (right hand), *LH* (left hand), *LA* (left-affected subject), *RA* (right-affected subject)

Spasticity, as measured by Modified Ashworth, showed modest correlations with a few task parameters: the number of objects hit with the right hand (*r*_*s*_ = −0.32, *P* = 4.2×10^−5^), movement area with the right hand (*r*_*s*_ = −0.34, *P* = 1.6×10^−5^), and hand speed with the right hand (*r*_*s*_ = −0.33, *P* = 2.6×10^−5^).

### Interrater reliability

The interrater reliability of the task parameters is shown in Table 2 for subjects (13 controls and 10 subjects with stroke) assessed in the task twice. Intraclass correlation coefficients were often high: ICC > =0.8, for 76 % of parameters. Lower reliability values were generally associated with parameters that identified fewer subjects with stroke as impaired and thus had a relatively small range of values across the control and stroke populations. In contrast, higher reliability values tended to be associated with parameters that identified more subjects with stroke as impaired and thus tended to have a larger range of values across the inter-rater sample.

## Discussion

The current study quantified impairments in stroke survivors to rapidly hit certain objects (targets) while avoiding all other objects (distractors). Up to 78 % of subjects with stroke had impairments in individual global, spatial, temporal, or hand-specific task parameters. The task instructions were simple, minimizing the impact of comorbid language impairment [39]. The task was completed in ~3 min yet provided a wide range of information related to sensorimotor and cognitive function. Most parameters had high inter-rater reliability providing an objective approach to measure impairments and track recovery.

The object hit and avoid task is a variant of an object hit task in which subjects had to rapidly locate and hit all objects moving in the workspace [22]. The present task extended this approach by requiring the subject to select amongst many options when moving and interacting in the environment. Total objects hit quantified each subject’s ability to make rapid motor actions, regardless of whether they hit the correct objects or not. Subjects with stroke almost always hit fewer objects with their more affected side, and this arm’s performance was more correlated with FIM scores than the unaffected side. Thus, the reduction and asymmetry of the ability to make rapid motor actions is quantitatively measured by the object hit and avoid task, and may have importance in the ability to complete activities of daily living.

We used a large number of parameters to quantify a broad range of sensory, motor and cognitive functions necessary to perform this task. For healthy subjects, some of these measures were highly correlated, but nevertheless captured different functions. For instance, the correlation between target hits and object hits was very strong for controls (r = 0.81). The reason why both parameters were measured rather than choosing only one was because it was important to differentiate between the ability to make fast and accurate movements, and the ability to make correct motor decisions om whether an object was a correct reach target or not. Thus, these metrics represent different domains of performance. Furthermore, subjects with stroke do not necessarily follow this typical pattern of performance. As shown in Fig. 2d, some subjects with stroke hit a high proportion of distractors and others do not, showing the value of each parameter to identify different impairments that do not necessarily co-occur in some individuals with stroke.

The inclusion of both target and distractor objects in the current task added an additional cognitive load to the previous object hit task. This is important as many different cognitive processes are necessary to perform daily activities, and their impairment after stroke is a significant cause of disability [40]. The present object hit and avoid task focused on a few key processes.

First, demands on the attentional system are high in a visual search task, as it requires differentiating target and distractor stimuli [41]. Rapid parallel processing of the entire visual workspace can be employed to find a target amongst many distractors with minimal effort if the target has a unique feature separate from distractors that makes it ‘pop out’. In contrast, focused attention is required to serially analyze each stimulus if the target can only be differentiated from the distractors by a conjunction of features. The greater attentional demands required for a conjunction versus a feature visual search task results in greater reaction time for both controls and subjects with stroke who do not have visuospatial neglect [23]. Subjects with visuospatial neglect also show significantly increased times to detect targets in a conjunction search task (regardless of which side of the workspace was tested), when compared to the performance of controls and subjects with stroke. The object hit and avoid task is representative of a conjunctive visual search as targets could only be differentiated from distractors by attending to the geometry (circular, three- or four-sided) and relative dimensions (tall, wide or equal) of each object (see Methods-Behavioural Task). Correspondingly, BIT scores correlated with many individual task parameters, as well as the total number of parameters impaired. Although correlations were weak to moderate, all were in the expected direction: greater task impairment associated with greater clinical impairment.

In the current study, participants are required to either enact a reach toward the target, or actively avoid hitting a distractor. Despite visual feedback, haptic feedback, and initial reminders on the need to hit only two types of objects and avoid the rest, over half of the subjects with stroke hit a greater proportion of distractors than 95 % of controls. Subjects with stroke were twice as likely to be impaired in this parameter if they also had neglect.

The ability to inhibit a motor action is an important cognitive function of voluntary motor behaviour [7]. Motor decisional processes mediate the initiation of an automatic motor response to a new stimulus with the voluntary response required by the task [42]. This ability to inhibit stimulus-driven and enact task-driven motor responses can be measured by eye movements in the anti-saccade task [43] and arm movements in an anti-pointing task [8]. In both tasks, subjects must inhibit a movement to the appearance of a visual stimulus and move to the equal and opposite location. Subjects with stroke having damage to frontal lobes have been shown to make erroneous saccades towards a stimulus in an anti-saccade task [9]. Subjects with stroke and visual neglect show greater endpoint errors and longer reaction times in an anti-pointing condition (on both sides of space) than controls or subjects with stroke who do not have visuospatial nelgect [24]. Distractor proportion in the current study correlated with BIT scores just as anti-pointing impairments correlated with the severity of neglect.

The assessment of rapid visuomotor skills post-stroke has potentially useful applications when rehabilitation goals are to regain high function. The object hit and avoid task may be very predictive of the ability to drive, return to work, or maintain complete independence as these skills require the ability to make many rapid motor decisions daily. We show that impairments in these skills are not always captured by currently used pen and paper cognitive screening tools such as the MoCA. Also, since this task relies on many domains of function to be successful, it may be a good indicator of overall stroke recovery.

The measurement of cognitive function after stroke, as measured by the MoCA, correlated moderately with distractor proportion, but only modestly with the number of distractor hits. As well distractor proportion also identified more subjects as impaired as compared to distractor hits. These differences reflect the fact that some control subjects hit a substantive number of distractors, but they also hit many targets. This is why we measured distractor proportion which quantified the ratio between distractors hit and total objects hit.

This task is also part of a larger research program to design a battery of robotic assessment tasks to create a quantitative diagnostic assessment of sensory, motor, and cognitive impairments post-stroke [21]. The use of a robotic assessment provides objective, continuous measures of performance that are responsive to small changes and compared to a normative model of healthy control performance. This overcomes issues of subjectivity, coarse ordinal scales, criteria-based scoring, and lack of responsiveness (including floor and ceiling effects) seen in many standard assessments of post-stroke functioning. We have also developed assessments of visually-guided reaching [44], bimanual control [30], limb position sense [26], kinesthesia [45], and limb afferent feedback for action [46]. The goal is that information from this assessment battery may be used collectively to provide more precise and responsive tools to guide individualized rehabilitation care.

Successful performance in the current task requires many sensorimotor and cognitive skills, thus failure can reflect many potential impairments in sensory, motor and cognitive functions. In order to identify unique impairments in individual participants, it is important to consider the type of parameters that show poor performance. For example, subjects who have impairments in the number objects hit, but not distractor proportion, may have underlying sensorimotor impairments, but no cognitive impairments. These subjects may be better candidates for sensorimotor rather than more cognitive-related rehabilitation. Future work is required to identify whether these patterns of impairment can predict the best type of rehabilitation for each individual.

## Conclusions

The object hit and avoid task provides a simple and fast approach to quantify the use of attention and selection to perform rapid motor actions with the arms. Most subjects with stroke were found to be impaired when performing this task, especially those with neglect. Many parameters had high inter-rater reliability and correlated with various clinical measures of impairments and ability to perform daily activities.

## Abbreviations

AA: Affected arm
BIT: Behavioural inattention test
Br: Brainstem
C: Cortical
C + SC: Cortical + subcortical
Cb: Cerebellar
Cb + Br: Cerebellar + brainstem
CMSA: Chedoke-McMaster stroke assessment
CR: Control range
DA: Dominant arm
FIM: Functional independence measure
L: Left
LA: Left-affected
LH: Left hand
M: Mixed
MoCA: Montreal cognitive assessment
Mx: Mixed
NDA: Non-dominant arm
R: Right
RA: Right-affected
RH: Right hand
SC: Subcortical
UA: Unaffected arm
Uk: (unknown)
